# Importance-performance analysis in fitness apps. A study from the viewpoint of gender and age

**DOI:** 10.3389/fpubh.2023.1226888

**Published:** 2023-08-14

**Authors:** Francisco Martín, Jerónimo García-Fernández, Manel Valcarce-Torrente, Ainara Bernal-García, Pablo Gálvez-Ruiz, Salvador Angosto-Sánchez

**Affiliations:** ^1^Physical Education and Sport Department, Universidad de Sevilla, Seville, Spain; ^2^Faculty of Law and Social Sciences, Valencian International University, Valencia, Spain; ^3^Department of Sport and Computer Science, Universidad Pablo de Olavide, Seville, Spain; ^4^Department of Physical Activity and Sport, University of Murcia, Murcia, Spain

**Keywords:** fitness apps, digital platforms, cluster analysis, gender, age

## Abstract

**Background:**

We are currently undergoing a profound process of digital transformation that has favoured the development and use of apps in multiple facets of people’s daily lives. In the fitness industry, this situation has facilitated the control of exercise and the maintenance of healthier lifestyles. However, it is not known how the perceived quality and importance of fitness apps vary for users based on gender and age, which is the objective of this study conducted among users of fitness centres.

**Methods:**

By means of a convenience sample, 321 users from different centres of the boutique fitness chain Sano Centre (238 females and 83 males) took part in the study. They answered the 16 items of the MARS scale, distributed in four dimensions, in terms of importance and perceived quality. The existence of significant differences was analysed using non-parametrical statistics such as the U-Mann-Whitney (gender) and the H-Kruskal-Wallis (age). In addition, a cluster analysis, combining hierarchical and non-hierarchical methods, was analysed considering as a dependent variable the level of recommendation of fitness apps.

**Results:**

Considering gender, in importance-performance analysis (IPA), credibility was the most important attribute for females and quality of information for males. In the case of age, credibility was the most important attribute in all the ranges. The cluster analysis established two groups (high and low recommendations of the fitness app). In importance, the first group scored better on all factors except entertainment and interest. In valuation, the scores were lower than on importance, especially in the low recommendation group.

**Conclusion:**

Regarding usage behaviour, credibility is the factor to which users attach the highest importance and rating, regardless of gender and age. The main demand focuses on improving the gestural design and visual appeal, which will facilitate a better user experience.

## 1. Introduction

Today’s society is immersed in a major process of digital transformation, accelerated by the global pandemic caused by COVID-19, in which technology and its different devices are here to stay. The lockdown imposed by the vast majority of countries led to a decrease in the levels of physical activity of the population, which began to use fitness apps to be able to exercise and combat psychological problems such as anxiety or depression (1). Analysing the current impact of technology on society, we are social (2) estimates that 67.1% of the world’s population has a mobile phone, 96.2% of which are smartphones. Furthermore, it also specifies that 92.1% of the population accesses the internet via a mobile device.

Digitalisation allows consumers a greater accessibility and increased interaction and control of products and services regardless of the access device (i.e., mobile, tablet, television, or computer). In addition, it enables the sharing of customer interests and needs, optimising resources in a more economical, efficient, and sustainable way, typically through Apps (3). In fact, Apps allow users to perform a multitude of functions and tasks related to organization, management, communication, and entertainment (4). Thus, according to App Annie (5), 230 billion Apps were downloaded in 2021, representing a revenue of 170 billion dollars. Specifically in the fitness industry, Sensor Tower (6) states that fitness and health-related app downloads in Europe in 2021 reached a total of 290 million downloads. Recent reports highlight that among health and fitness apps, those related to physical activity and fitness are among the top ten apps in terms of downloads and spending in recent years (6, 7). However, a recent review found that there is a huge gap in the market between the existing offer and the needs of fitness app users, with much potential for improvement (8).

The main function of these Apps is to send and receive data through the Internet of Things or other users (9). Moreover, considering existing theories of technology use behaviour, they provide information to analyse human behaviour by adapting the services offered to the needs of their users (10). In fact, recently Caparrós et al. (11) affirmed the importance of technology in a greater efficiency and degree of compliance with physical activity programmes if Apps were used.

Therefore, technological advances have made it possible to facilitate the formation and maintenance of healthier lifestyles through a greater control of physical exercise via an App. Likewise, the fitness industry has become a global business that is progressively expanding at all levels in which managers and fitness centres must renew themselves through a continuous digital transformation. Thus, the popularity of fitness Apps among users is so great that predictions estimate that they will reach revenues of around 13 billion dollars in 2025, i.e., an increase of 134% compared to 2020 (12). Moreover, in parallel to this technological development, the scientific literature linked to fitness Apps has been evolving, considering aspects such as behavioural theories, technological characteristics, human behaviour concerning health, and the social influence surrounding users who employ this type of Apps. However, it has not been studied which are the functions and characteristics that users of this technology value and give importance to. In fact, the perceptions according to gender and age, and therefore the specifications in line with different population niches, are so far unknown. In this sense, the aim of this study is to find out the perception and importance of fitness Apps according to gender and age in users of fitness centres.

### 1.1. The perceived quality in fitness apps

The perceived quality of an App is complex to establish as it can be a subjective aspect and linked to a personal opinion when users evaluate it. Montazami et al. (13) state that to measure the perceived quality of Apps it is necessary to take into account general and specific aspects shared by Apps in the same field (for example, all those focused on fitness and health).

The concept of “perceived quality” associated with technologies does not have a definition that clearly contemplates objectivity as it is a personal opinion that depends on various factors (14). Considering various definitions of this concept, within the technological field in general it can be understood as the evaluation of the content, strategies and technical and functional characteristics offered by digital tools and devices to users (15, 16). In the context of Apps (app store), perceived quality “refers to the app’s user interface design and the performance and stability of the software program” (17), p. 1052. Similarly, the perceived quality of Apps can be understood as an overall assessment of the different criteria proposed by the users themselves and the performance of the App with respect to these established criteria (18). The importance of the perceived quality of the Apps lies in the fact that if it is perceived positively, users will show a greater intention to download it and to use it in the future (19). In turn, and in the case of fitness Apps, this perception of the App could be linked to factors such as the schedules of sports activities, the supervision by the virtual trainer, the content of the sessions, or an approach to fun and entertaining workouts (20).

Regarding the instruments used to measure the perceived quality of Apps, the proposal by Stoyanov et al. (21), who developed the Mobile app Rating Scale (MARS), an instrument that assesses the perceived quality of health Apps, stands out. This tool makes it possible to evaluate health Apps in a reliable and multidimensional way (a characteristic that it shares with the concept of perceived quality), moving away from the scoring system based on the stars of user reviews. While this tool has been used in different contexts, recently Roberts et al. (22) used it to evaluate health Apps and found it useful in guiding health professionals in their exploration of apps for clinical use. Among their results, they found that aspects such as training for use, time or skill of use could become facilitators for the perceived quality.

In turn, this tool has also been used in fitness Apps, such as the study by Park et al. (23), where they found that relevant elements related to the functionality of fitness Apps were self-efficacy, innovativeness, outcome expectancy and engagement. Also, Paganini et al. (24) concluded that the perceived quality of fitness Apps depends on the importance of objective quality, such as engagement, functionality, aesthetics and information quality, and the subjective quality of the user. However, it has to be taken into account that according to the World Health Organisation (25), health Apps, including fitness Apps, should be accessible to everyone and therefore those of any gender, age or culture. As a result, the analysis of the perceived quality of the Apps could shed light on their accessibility (26). In addition to perceived quality, other variables have been shown to be influential for users to utilise them. In fact, Damberg (27) found multiple factors that could influence fitness app usage intention such as health consciousness, habit, perceived enjoyment, performance expectancy, and price value.

### 1.2. The perceived quality of fitness apps by gender and age

It is also important to note that within the marketing and scientific literature, the different variables have always been analysed according to different socio-demographic factors, especially gender and age. On the one hand, gender has always been used to target the population due to the differences in opinion and perception that often exist between males and females (28, 29). On the other hand, the age factor has become a determining factor in recent years due to the digital divide, which is manifested in the lower use of technology in middle-aged and older adult audiences (30). Finally, the Unified Theory of Acceptance and Use of Technology 2 (UTAUT2) model suggests that both gender and age are two contextual factors that influence technology adoption and use (31).

In the sport context, gender and age are key factors for the use of fitness Apps, mainly because of the importance of people’s characteristics (32), in addition to other factors, such as facilitating conditions or habit. The literature reviewed on perceived quality shows that gender is a factor affecting consumer behaviour. Several studies found that males are more likely to use fitness apps than females (33, 34). With the growth of Apps and social media in recent years, gender has become an influential variable in global marketing strategies (35). Although there is a lack of research on the subject, it is known that users with greater knowledge about a field or sector are more aware of its perceived quality (36). In addition, these authors found that males perceived their favourite brand as having a better perceived quality compared to females. However, females were more interested in perceived quality as a concept, seeking more information to evaluate it.

Oyibo et al. (37) found differences between males and females in App responsiveness, where females were less responsive to rewards and cooperation compared to males. Another difference found was that males were more likely to be motivated by competitive physical activities compared to females (38). Therefore, depending on the target audience of the fitness App, those factors that have more persuasive power than others should be taken into account, such as cooperation, which will be a factor that mainly motivates females, while for males’ competition will be more motivating. However, Baer et al. (30) recently claimed that the existence of gender differences might depend on the App itself and therefore would not be a general difference for all people. In terms of information and system quality, Park et al. (39) found gender differences where females are more concerned about safety and the potential risk while using technologies.

In another study Junker et al. (40) assessed the intention to use health promotion apps at work, finding gender differences, with females having a higher normative belief in the apps than males. On the other hand, females were welcomed gamification features more in fitness apps than males (8). Yet, considering the COVID-19 pandemic period, both females and males showed similar factors in fitness app usage during the lockdown (41). However, these authors indicated that females had more influential moderating factors than males with respect to affiliation, security and privacy risks. For these reasons, the gender gap in the adoption of new technologies via smartphones is decreasing, although both genders have different motives in the process of adopting the technology (42). In fact, there are also studies that reported no gender differences in technology adoption (27, 30, 43, 44).

Regarding age, Yu et al. (45) stated that the perceived quality is linked to age and that it allows intervening in attracting, maintaining and increasing the number of customers. This is justified on the grounds that the older the users, the more experienced, knowledgeable, better planned and treated they are as customers. Therefore, this transforms them into more demanding and critical people in their sports consumption, determining a value of the perceived quality which is different from that of younger users (46). In particular, middle-aged and older adults have a lower acceptance of fitness apps than younger users (30). This is because middle-aged and older people are less interested in using apps as they perceive them as not very useful (47).

In fact, it should be kept in mind that most fitness Apps are geared towards young adults (48). Thus, Oyibo et al. (37) found that, following the use of Apps, younger people (less than 24 years old) were more receptive to competitiveness, comparison and social learning in contrast to older people (24 years old or more). Similarly, Shih and Jheng (49) stated that age influenced the reception of persuasive strategies used in fitness Apps, and therefore their perceptions vary across age groups. For example, rewards as a strategy to continue using the App were more persuasive for young adults (under 40 years) than for older adults (40 years or more). Also, young users are more receptive to gamification features in fitness apps than older adults (8), while young people under the age of 25 have a higher perceived usefulness concerning fitness app usage intention than users over the age of 30 (50).

However, self-monitoring and cooperation in fitness Apps were more persuasive factors for older adults. Another interesting finding was social comparison, which was more motivating for younger people but had the opposite effect on older people (51). Thus, it could be said that age is a factor in the intention to use technologies and therefore in the perceived quality of fitness Apps (52). So, an effort should be made to guide studies that analyse the needs and perceptions of fitness Apps according to age (30). However, other studies such as that of Chiu et al. (44) did not find age to be a determining factor in the intention to remain using the fitness app.

Therefore, although gender and age have been found to influence the perceived quality of technology and especially Apps, the long-term appropriation of these devices is subject to many conditions, such as the acceptance of the App standards or the ability to do sport. However, it should be noted that people who consistently use fitness Apps have a more active lifestyle and therefore maintain the use of Apps (53). In other words, regular users of fitness Apps are generally already physically active and are long-lasting users (54). In contrast, for other users, the success of these Apps is the change of behaviour to a healthier or more physically active habit. For example, if abandonment of the App occurs, this could lead to disengagement from the adopted behaviour (55). Thus, determining the perceived quality of the App’s features could influence further use by both current and potential future consumers.

In summary, some of the other factors that have been found to be determinants beyond gender and age indicate that fitness app users with a higher level of income or social status are more likely to use fitness apps (27, 33, 34), while people who are overweight or obese have a better consideration of the app’s gamification function (8). The size of the social network also influences how users rate themselves and the app (56). In relation to health, Kim and Han (43) found that health status is not a differential factor for physical activity through health apps.

### 1.3. The importance-performance analysis

The importance-performance analysis (IPA) is an underutilised method in the literature on technology and perceived quality (57, 58). The IPA developed by Martilla and James (59) is a method or technique for measuring consumer perception by analysing the reality sensed by consumers compared to their expectations. This method uses two dimensions (importance and valuation) to provide information on those aspects that require improvement and which have a direct effect on user satisfaction, or on their use in the case of Apps (58). According to Rial et al. (60), the IPA method is an approach for measuring user perception that allows a simple and functional identification of strengths and weaknesses, or areas for improvement. In addition, it also provides an insight into areas where resources are over-invested, elements where resources should be maintained, or those that are of no relevance to the user (61).

The IPA diagram consists of four quadrants, in which the different dimensions that comprise it are established, where the vertical axis represents the values of importance and the horizontal axis the values of valuation (62). The quadrants identified in the IPA are: quadrant I determines those elements with a relatively high level of importance but whose performance is low. Here the elements need to improve their performance. Quadrant II determines those elements with a relatively high level of importance and a relatively high level of performance. The items in this quadrant are considered as a factor supporting user satisfaction and should be maintained. Quadrant III determines the items with a relatively low level of importance and a relatively low level of performance. Items that fall into this quadrant have a low priority. Quadrant IV determines items with a relatively low level of importance and relatively high performance. Elements in this quadrant may indicate a possible excess of resources.

This analysis technique has been used in marketing strategies to analyse and compare those aspects that are of high importance in the services provided to consumers in fitness centres (63, 64). In this way, it allows a very intuitive visual assessment of fitness centre management and corresponding advice for a better allocation of organizational resources. However, it is not known which aspects are the most important and highly valued by fitness app consumers, even though we are aware of the increased use of these digital tools (65). Thus, trainers, managers and developers could know more precisely the needs of their consumers in order to improve, maintain and encourage the use of fitness Apps.

A recent study found that males had perceived enjoyment as a predictor and the most prominent exogenous variable was system quality (58). For females, perceived enjoyment was the most predictive factor and the most influential exogenous variable. Regarding the IPA analysis, Won et al. (58) found that the relationships of the quality dimensions (information and quality) and the determinants of the technology acceptance model (perceived usefulness, perceived ease of use and perceived enjoyment) varied between males and females in predicting users’ intention to use fitness apps.

Finally, Grundy et al. (17) concluded the need to use innovative methods that evaluate the content and functions of fitness Apps and which are currently successful for the consumers who use them. Therefore, the use of this technique in fitness Apps could help, on the one hand, to establish better digital tools, and, on the other hand, to encourage their use and therefore the promotion of healthy habits. Consequently, gender and age have been found to be two key determinants of perceived fitness app quality and intention to use. The UTAUT2 is a theoretical model being increasingly more used by expert authors in the field when assessing the adoption and use of technologies, specifically fitness apps. Venkatesh et al. (31) highlighted that the main determinants of attitude and intention to use technologies in addition to gender and age were user experience and willingness. Hence, further contributions to the literature related to IPA analysis and perceived quality of fitness app according to these determinants are needed.

## 2. Methodology

### 2.1. Data collection

Using a convenience sampling technique, the sample included a total of 321 participants, specifically 238 females and 83 males. With regard to the age, similar percentages of participation were obtained in both genders in all the age ranges used, concentrating the largest volume of participants between 31 and 40 years, as well as between 41 to 50 years (Table 1).

**Table 1:** Age range of the participants.

	Women	Men	Total			
	*n*	%	*N*	%	*n*	%
*Age (years)*						
From 17 to 30	46	19.33	9	10.84	55	16.98
From 31 to 40	78	33.19	32	39.76	113	35.49
From 41 to 50	78	33.19	30	36.14	108	33.64
From 51 to 65	34	14.29	11	13.25	45	13.89
*Total*	236	73.46	82	25.62	321	100

### 2.2. Survey instrument

The questionnaire used for this research was the MARS scale (21), a tool adapted and validated in Spain by Martín-Payo et al. (66), showing adequate psychometric properties to assess the perceived quality of health Apps from the user perspective. The scale is composed of 16 items distributed in four dimensions to evaluate in this study the perceived quality and importance of the elements of the Fitbe^®^ App: commitment (five items), functionality (four items), aesthetics (three items) and information (four items) (Table 2). The response format for all the items was a 5-point Likert scale. To evaluate the importance, the Likert scale ranged from not at all important (1) to really important (5), and in the case of perceived quality, responses ranged from totally disagree (1) to completely agree (5). In the first place, we asked about the importance that each item had for the participants, and then, in the same order as the previous ones, about the perceived quality that they gave to each item.

**Table 2:** Dimensions and items of the uMARS scale.

Item	Dimension and item
*Commitment*	
1	Entertainment
2	Interest
3	Personalization/Customization
4	Interaction
5	Targed audience
*Functionality*	
1	Performance
2	Ease of use
3	Navigation
4	Gesture design/Gestural design
*Aesthetic*	
1	Provision/Disposal
2	Graphics
3	Eye appeal/Visual appeal
*Information*	
1	Quality of information
2	Quantity of information/Amount of information
3	Visual information
4	Credibility

### 2.3. Procedure

The data were collected during 1 month, after obtaining the approval of the chain of personal training centres “Sano Centre” (currently it has 60 fitness centres in Spain), to send the proposal to participate in the study to the customers. The choice of the Sano Centre chain was motivated by the fact that it is one of the fitness centre chains with the largest number of sports facilities in Spain, and with the greatest personalisation of the sports service. In turn, this chain was chosen because its services include the use of Fitbe, which is the fitness App that was analysed. Fitbe is a software as a service linked to a fitness App whose main features are the booking of a sports activities agenda, the administrative management of users, the monitoring and control of sport training, the nutritional control, and the management of gamification actions. For data collection, a Google Form was built with all the items, and Sano Centre was in charge of sending it to its customers. Prior to completing the questionnaire, informed consent was obtained from the participants after initially reading the description of the study, and the confidentiality and anonymity of their responses were guaranteed. After consent was accepted and the objectives of the study were explained, the participants agreed to answer the questionnaire. The questionnaire took about 7–8 min to complete.

### 2.4. Data analysis

The IPA analysis was used to identify the values of the importance and the perceived quality of the different items in terms of the general mean, the discrepancy, and the segmented values according to the age range and gender. In the case of the discrepancy (difference between two figures that should be the same, but are obtained from different information sources), this was calculated using the difference in the score between importance and perceived quality. The representation was carried out through a scatter plot of points in a biaxial representation (*y* axis: importance; *x* axis: perceived quality), where each item measured in the quadrant was positioned, determining the importance-performance of each of them according to the IPA analysis.

The normality of the data was tested by univariate skewness and kurtosis, without values smaller than the criteria 3 and 7, respectively (67). The results showed the no parametric data of the variables. A comparative analysis was performed to observe possible significant differences for both gender (U-Mann–Whitney test) and age variables (Kruskal–Wallis test). The significance level was set at a value of *p* < 0.05.

Finally, a cluster analysis was carried out to identify possible groups of fitness App users with similar opinions about the perceived quality and the IPA analysis of a fitness App, taking as a dependent variable, the item ‘Recommendation of the fitness App to others’ (66). To obtain the cluster solutions, two methods were combined, hierarchical and non-hierarchical, with the aim of optimising the results. The cluster analyses were carried out using the guidelines proposed by Romesburg (68). The hierarchical cluster was analysed employing Ward’s Method, and for the similarity measures the Euclidean distance squared was used. Then, a non-hierarchical cluster was performed through the *K-means method*, taking as a reference the centroids of the cluster solutions of the hierarchical method for each period. All the data were analysed with IBM SPSS version 22.0.

## 3. Results

### 3.1. Discrepancy analysis

As a first step, a discrepancy analysis was carried out using a global sample, as well as according to gender and age ranges (Table 3). Considering the global sample, the greatest discrepancies were observed in the functionality and information dimensions (2 items each dimension), specifically, ease of use (−0.48), and navigation (−0.47) of the functionality dimension, and quality of information (−0.44), and visual information (−0.40) of the information dimension. The most positive discrepancies were for 4 of the 5 items in the commitment dimension: entertainment (0.94), personalisation (0.21), interest (0.17), and interaction (0.10).

**Table 3:** Discrepancy analysis.

Items	General	Female	Male	17–30 years	31–40 years	41–50 years	51–65 years
*Commitment*							
Entertainment	0.94	1.05	0.70	0.56	0.77	1.16	1.29
Interest	0.17	0.29	−0.11	0.13	−0.10	0.41	0.31
Personalization/Customization	0.21	0.29	0.07	0.40	0.00	0.25	0.40
Interaction	0.10	0.17	−0.08	0.20	−0.05	0.18	0.16
Targed audience	0.05	0.01	0.24	−0.16	0.03	0.10	0.27
*Functionality*							
Performance	−0.15	−0.13	−0.18	−0.45	−0.23	0.06	−0.09
Ease of use	−0.48	−0.44	−0.51	−0.64	−0.57	−0.30	−0.47
Navigation	−0.47	−0.44	−0.47	−0.58	−0.68	−0.31	−0.18
Gesture design/Gestural design	−0.32	−0.28	−0.35	−0.55	−0.31	−0.26	−0.24
*Aesthetic*							
Provision/Disposal	−0.10	0.03	−0.37	−0.11	−0.03	−0.22	0.04
Graphics	0.01	0.00	0.16	0.00	0.03	−0.10	0.22
Eye appeal/Visual appeal	−0.30	−0.19	−0.48	−0.24	−0.45	−0.28	0.00
*Information*							
Quality of information	−0.44	−0.41	−0.47	−0.35	−0.45	−0.44	−0.49
Quantity of information/Amount of information	−0.24	−0.20	−0.29	−0.07	−0.24	−0.35	−0.18
Visual information	−0.40	−0.34	−0.47	−0.42	−0.53	−0.28	−0.33
Credibility	−0.33	−0.35	−0.27	−0.25	−0.37	−0.30	−0.36

### 3.2. Importance-performance analysis by gender

**Figure 1:**
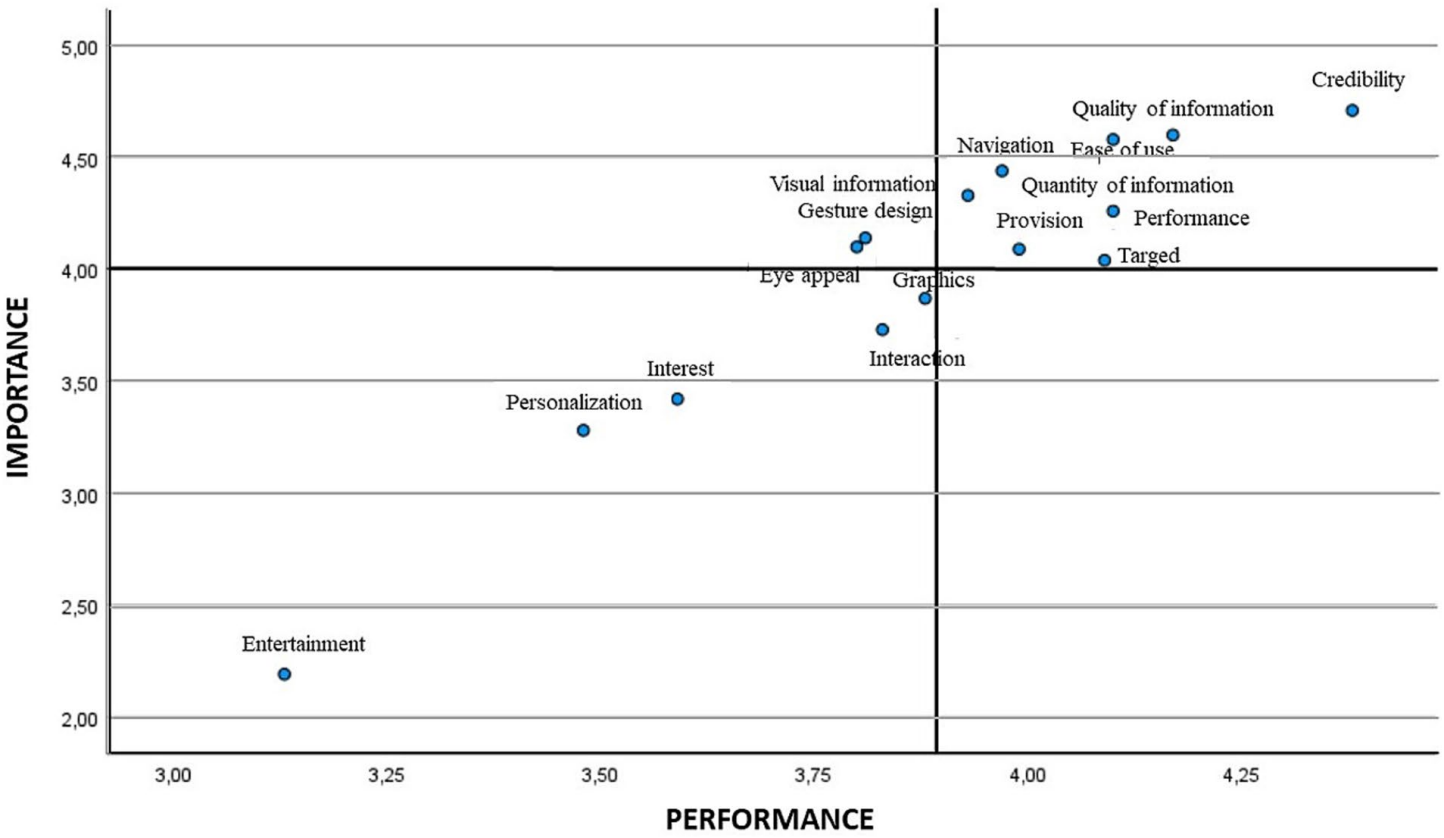
IPA Matrix general.

Next, an IPA analysis was carried out, thus verifying both the importance and performance of the different items of the questionnaire (Figure 1). To do this, the mean score was used, obtaining with the global sample the lowest importance and performance in item entertainment (2.19 and 3.13, respectively), with the highest mean score corresponding to item credibility for both importance and performance (4.71 and 4.38, respectively). When performing the analysis segmented by gender, for both females and males, entertainment was the attribute with the least importance and performance. As for the attribute that showed the greatest importance, for females this was credibility, while for males it was quality of information, although with a slightly higher value than credibility; regarding the performance, credibility was the highest attribute in both genders (Table 4).

**Table 4:** Importance and performance scores by gender.

Items	Total		Women		Men		U-Mann–Whitney	
	Imp.	Perf.	Imp.	Perf.	Imp.	Perf.	Imp.	Perf
*Commitment*	3.33	3.62	3.32	3.68	3.32	3.48	9737.5	8018.5**
Entertainment	**2.19**	**3.13**	**2.15**	**3.20**	**2.27**	**2.06**	9293.5	8856.5
Interest	3.42	3.59	3.37	3.66	3.53	3.42	9131.5	8642.5
Personalization/Customization	**3.28**	**3.48**	**3.25**	**3.54**	**3.29**	**3.36**	**9741.0**	**8918.0**
Interaction	3.83	3.83	3.73	3.90	3.72	3.64	9831.5	8518.5*
Target								
audience	4.04	4.09	4.12	4.13	3.77	4.01	8378.5*	8777.5
*Functionality*	4.35	4.00	4.40	4.08	4.20	3.82	8393.5*	8046.0*
Performance	4.26	4.10	4.30	4.17	4.12	3.94	8856.0	8578.0*
Ease of use	**4.58**	**4.10**	4.63	4.19	4.41	3.90	8809.5	8595.0
Navigation	4.44	3.97	4.50	4.06	4.25	3.78	8629.5*	8347.5*
Gesture design/Gestural design	4.14	3.81	4.18	3.89	4.00	3.65	8973.5	8523.0*
*Aesthetic*	4.02	3.89	4.06	4.01	3.85	3.62	8299.0*	7442.5***
Provision/Disposal	**4.09**	**3.99**	4.09	4.12	4.05	3.67	9534.5	7639.0***
Graphics	**3.87**	**3.88**	3.98	3.97	3.52	3.67	7690.0**	8333.0*
Eye appeal/Visual appeal	4.10	3.80	4.12	3.93	3.99	3.51	9271.5	7798.0**
*Information*	4.49	4.13	4.52	4.20	4.33	3.96	8793.5	7943.0**
Quality of information	**4.60**	**4.17**	**4.63**	**4.21**	**4.53**	**4.06**	9474.5	8918.5
Quantity of information/Amount of information	4.27	4.03	4.32	4.12	4.11	3.82	9222.0	8478.0*
Visual information	4.33	3.93	4.37	4.03	4.18	3.71	8776.5	8214.0*
Credibility	**4.71**	**4.38**	**4.78**	**4.43**	**4.51**	**4.24**	8323.0**	**8763.5**

Highlights in bold; Imp, importance; Perf, performance. *p < 0.05, **p < 0.01, and ***p < 0.001.

After the global analysis, the results for the different factors based on gender were verified, although it is important to consider the difference in samples. Significant differences were found in functionality and aesthetics, both for importance and performance, while commitment and information were only so in performance (*p* < 0.05). Considering the items, for the importance scale significant differences were obtained in the target audience and credibility items, while in the performance scale the differences were located in the items interaction, performance, gesture design/gestural design, provision/disposal, eye appeal/visual appeal, quantity of information/amount of information, and visual information. In addition, in both scales (importance and performance) there were significant differences in the items navigation and graphics.

**Figure 2:**
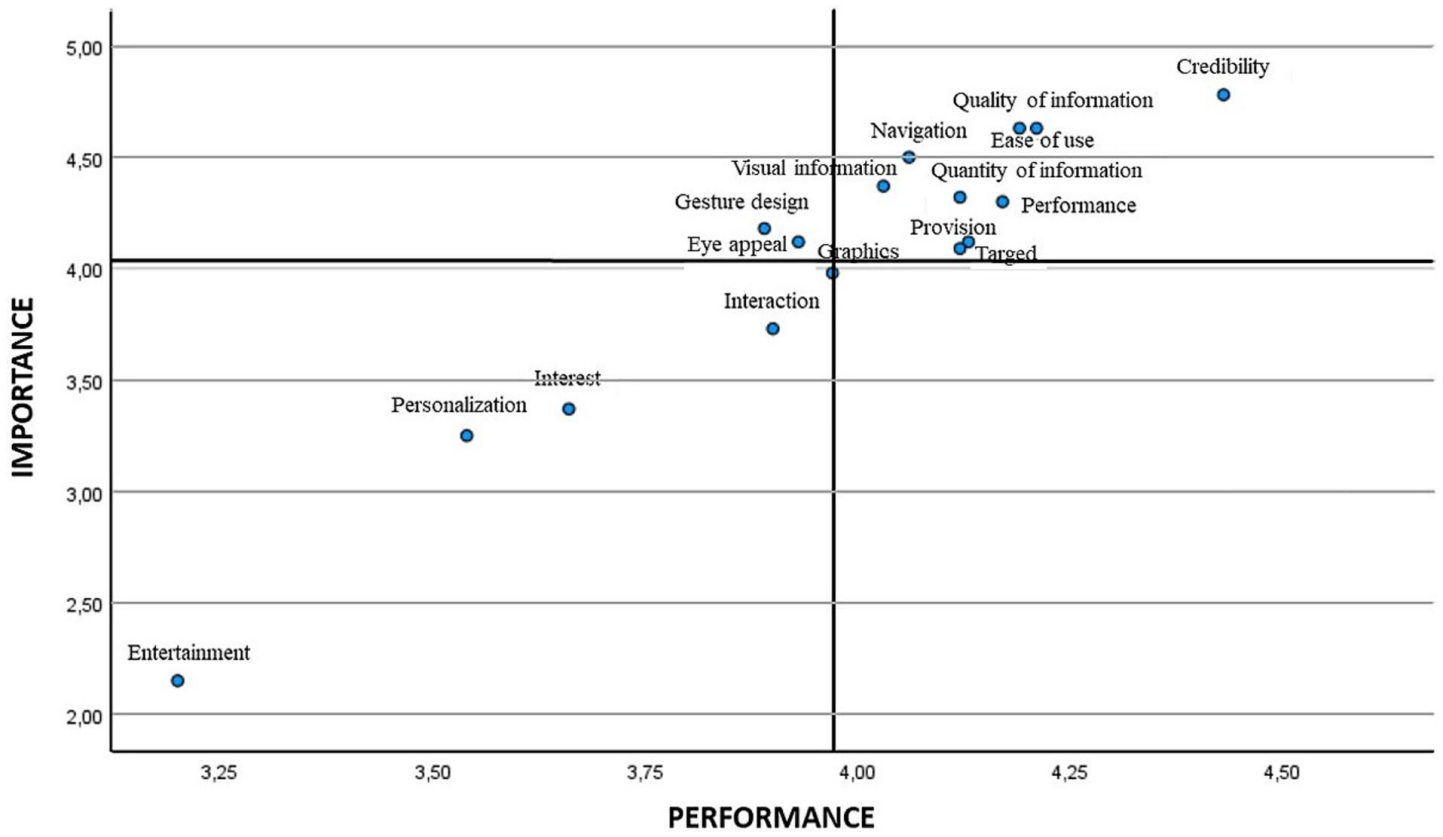
IPA matrix according to gender. Women.

**Figure 3:**
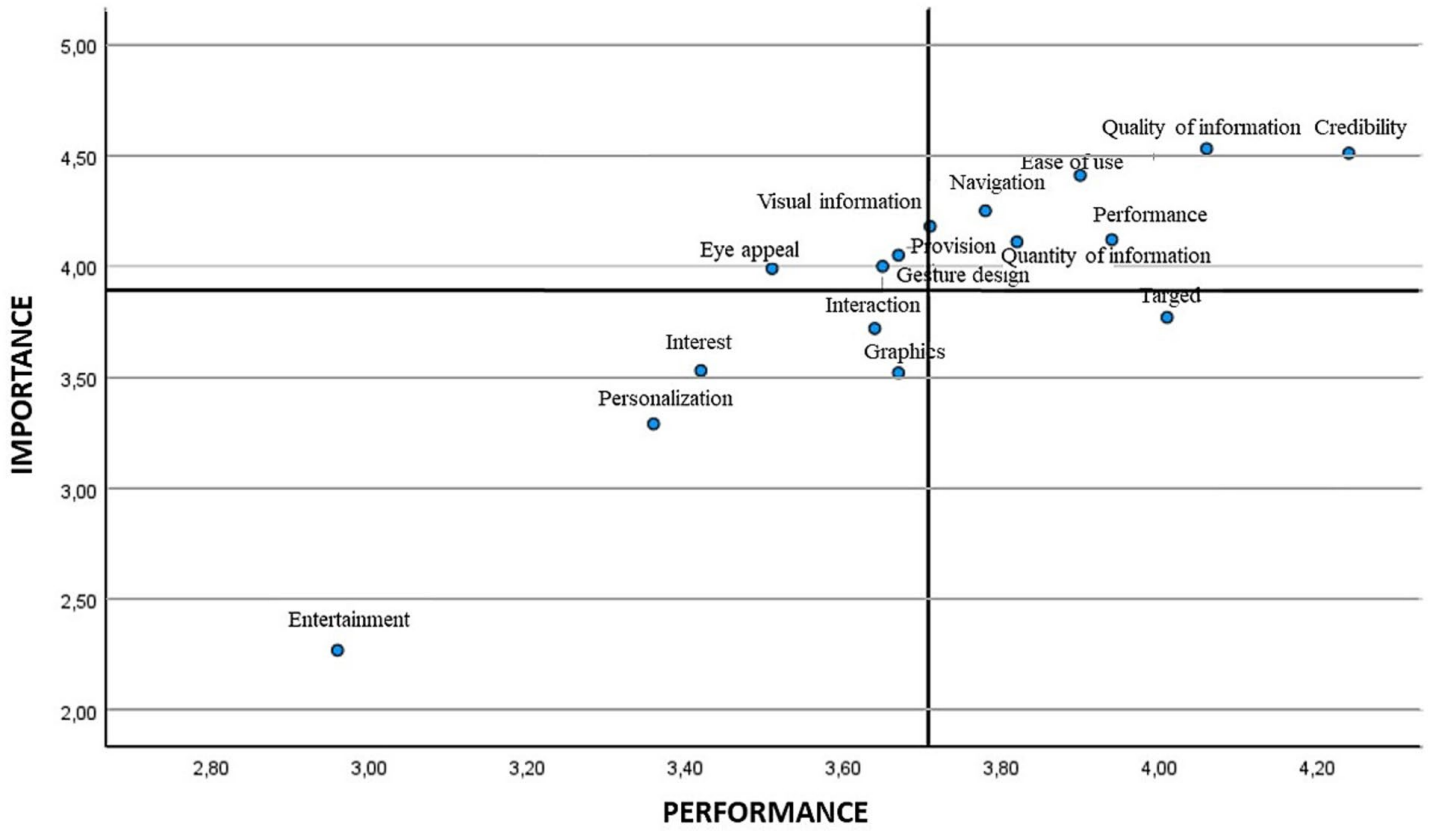
IPA matrix according to gender. Men.

Later, the means were employed for positioning the different attributes in the scatter plot. Figures 2, 3 show the IPA matrix according to gender. In the case of low-priority attributes, entertainment, personalisation/customisation, interest and interaction were obtained for both genders, although graphics were also found for males. The attributes of high importance and low satisfaction were eye appeal/visual appeal and gesture design/gestural design in both genders, in addition to provision/disposal in the case of males.

### 3.3. Importance-performance analysis by age

The last step was to check the IPA analysis and the data matrix based on the different age ranges used. The entertainment attribute is the one that shows the lowest score both in importance and performance for all ranges, except for the range of 51–65 years (personalisation/customisation). The highest score obtained is focused in all the cases on the credibility attribute (See Table 5).

**Table 5:** IPA analysis and the data matrix based on the different age ranges used.

Items	17–30 years		31–40 years		41–50 years		51–65 years		H-KW(df)	
	Imp.	Perf.	Imp.	Perf.	Imp.	Perf.	Imp.	Perf.	Imp.	Perf.
*Commitment*	3.41	3.64	**3.33**	**3.46**	**3.25**	**3.67**	**3.44**	**3.92**	1.50(3)	15.00(3)**
Entertainment	**2.24**	**2.80**	**2.17**	**2.94**	**2.09**	**3.25**	**2.44**	3.73	**3.15(3)**	15.61(3)***
Interest	**3.33**	**3.45**	**3.50**	**3.40**	3.28	3.69	3.67	3.98	3.51(3)	12.34(3)**
Personalization/Customization	3.40	3.80	**3.27**	**3.27**	3.26	3.50	**3.20**	**3.60**	0.68(3)	7.55(3)
Interaction	**3.95**	**4.15**	**3.71**	**3.66**	3.61	3.79	3.84	4.00	2.86(3)	11.54(3)**
Target audience	4.15	3.98	4.00	4.03	4.04	4.14	4.02	4.29	0.47(3)	4.51(3)
*Functionality*	4.40	3.84	4.37	3.92	4.30	4.10	4.40	4.16	0.272(3)	5.689(3)
Performance	4.42	3.96	4.29	4.05	4.15	4.20	4.27	4.18	4.47(3)	1.97(3)
Ease of use	4.58	3.95	4.60	4.03	4.51	4.21	4.69	4.22	1.73(3)	4.32(3)
Navigation	4.38	3.80	4.50	3.83	4.43	4.12	4.38	4.20	1.50(3)	8.19(3)*
Gesture design/Gestural design	4.20	3.65	4.08	3.77	4.12	3.86	4.27	4.02	1.61(3)	3.73(3)
*Aesthetic*	3.95	3.84	4.01	3.86	4.04	3.84	4.06	4.15	0.994(3)	6.499(3)
Provision/Disposal	4.04	3.93	4.03	4.00	4.18	3.96	4.07	4.11	1.74(3)	1.63(3)
Graphics	3.85	3.85	3.82	3.85	3.92	3.82	3.91	4.13	0.50(3)	5.21(3)
Eye appeal/Visual appeal	3.96	3.73	**4.18**	**3.73**	4.03	3.74	**4.20**	**4.20**	**3.15(3)**	8.33(3)*
*Information*	4.49	4.21	4.49	4.09	4.44	4.10	4.53	4.19	1.310(3)	3.614(3)
Quality of information	**4.67**	**4.33**	**4.62**	**4.17**	**4.55**	**4.11**	**4.62**	**4.13**	1.57(3)	3.99(3)
Quantity of information/Amount of information	4.16	4.09	4.29	4.04	4.29	3.94	4.31	4.13	1.28(3)	1.87(3)
Visual information	4.38	3.96	4.35	3.82	4.22	3.94	4.47	4.13	4.21(3)	4.21(3)
Credibility	**4.73**	**4.47**	**4.70**	**4.32**	**4.71**	**4.40**	**4.73**	**4.38**	**0.78(3)**	**1.25(3)**

Highlights in bold; Imp. = importance; Perf. = performance; H-KW=H-Kruskal-Wallis. * p < 0.05; **p < 0.01; ***p < 0.001.

Analysing the possible differences between age groups, it was observed that there were statistically significant differences only in the performance scale (*p* < 0.05). Specifically, statistically significant differences were found in the interaction item between the 17–30 and 31–40 age groups. Also, statistically significant differences were found in the entertainment, and navigation items between the 17–30 and 51–65 age groups. On the other hand, the 31–40 age group had statistically significant differences to the 51–65 age group concerning the commitment factor and the entertainment, interest and eye appeal/visual appeal items (*p* < 0.05).

**Figure 4:**
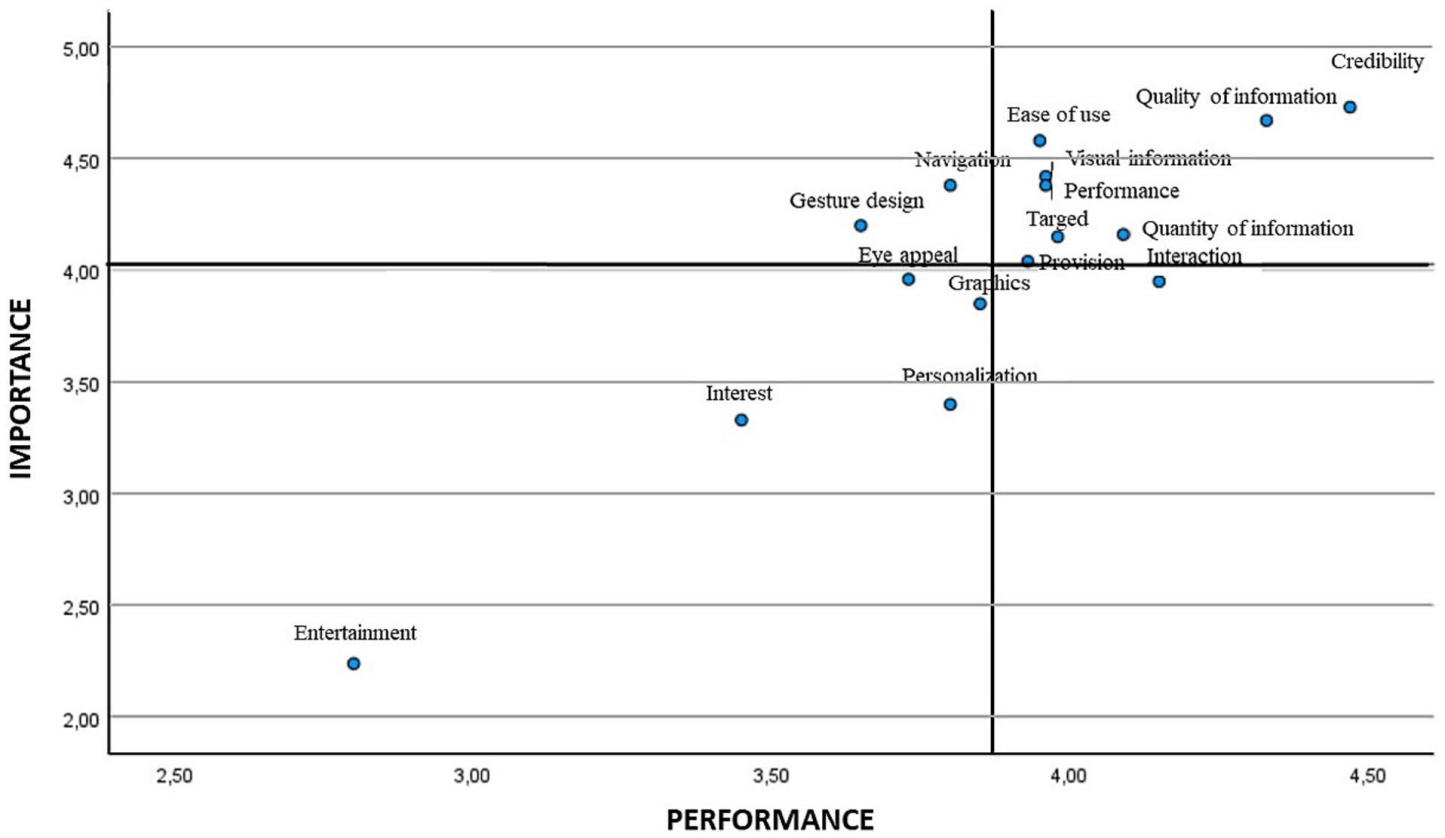
IPA analysis in the age range of 17–30.

**Figure 5:**
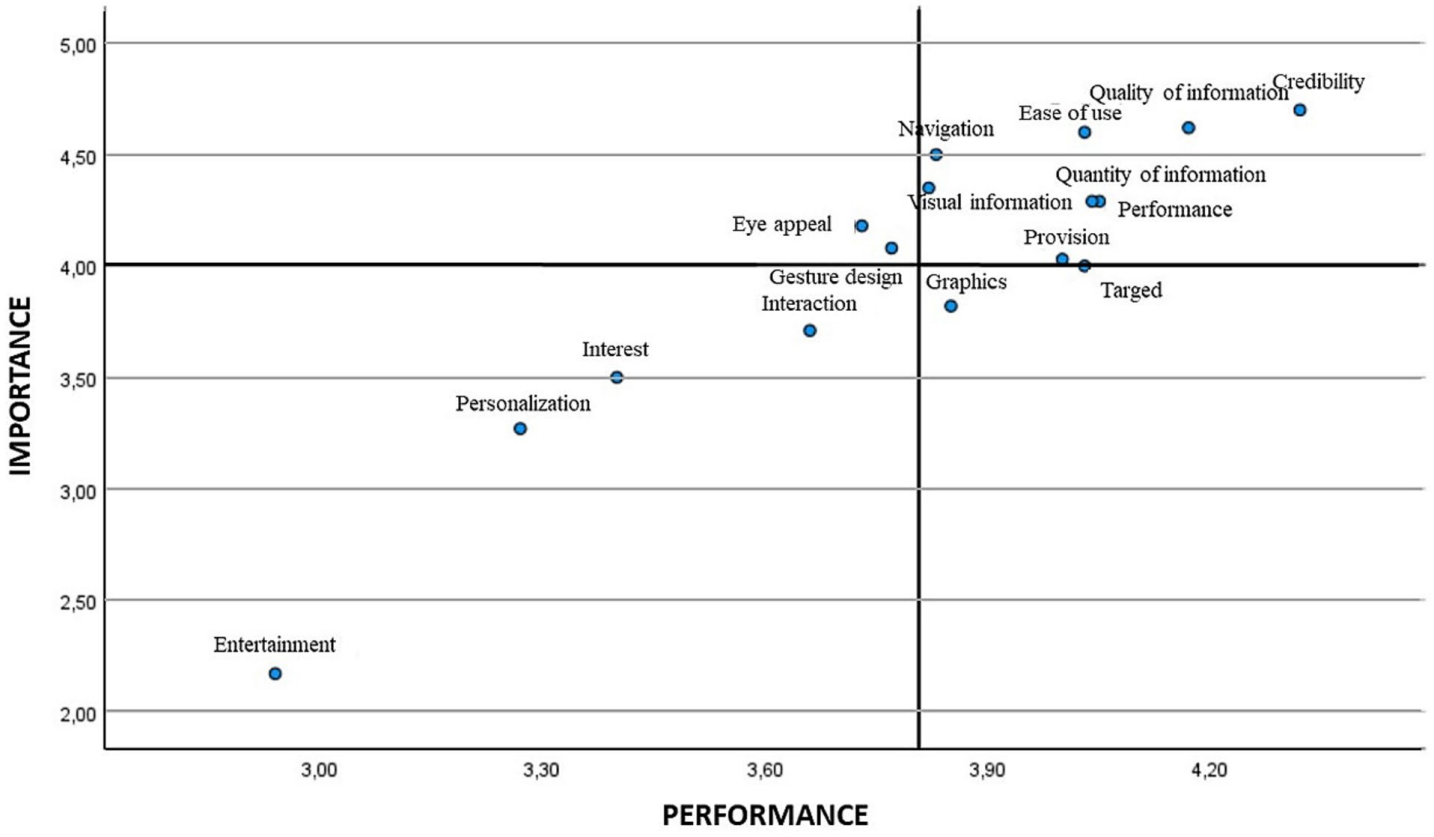
IPA analysis in the age range of 31–40.

**Figure 6:**
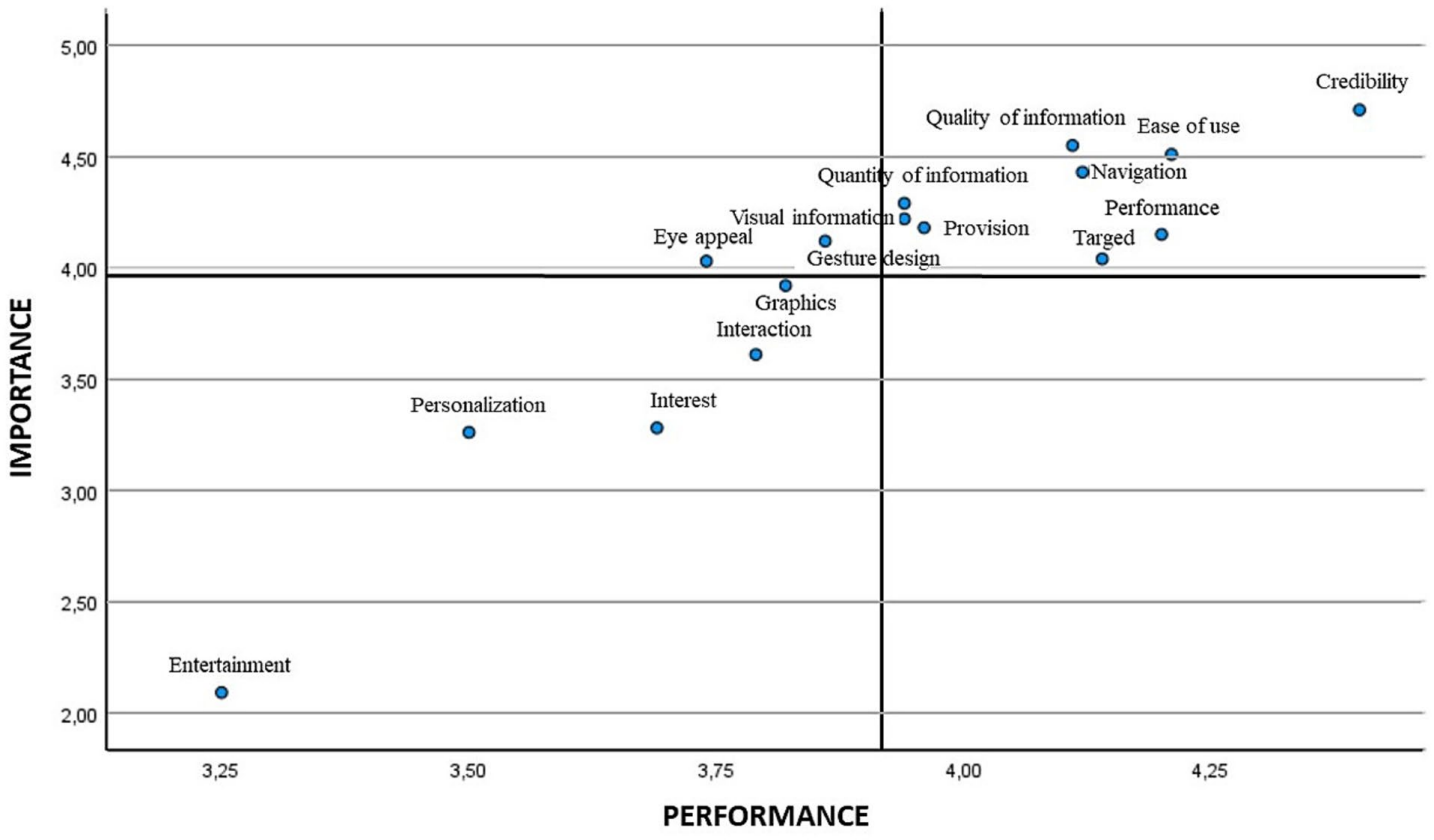
IPA analysis in the age range of 41–50.

**Figure 7:**
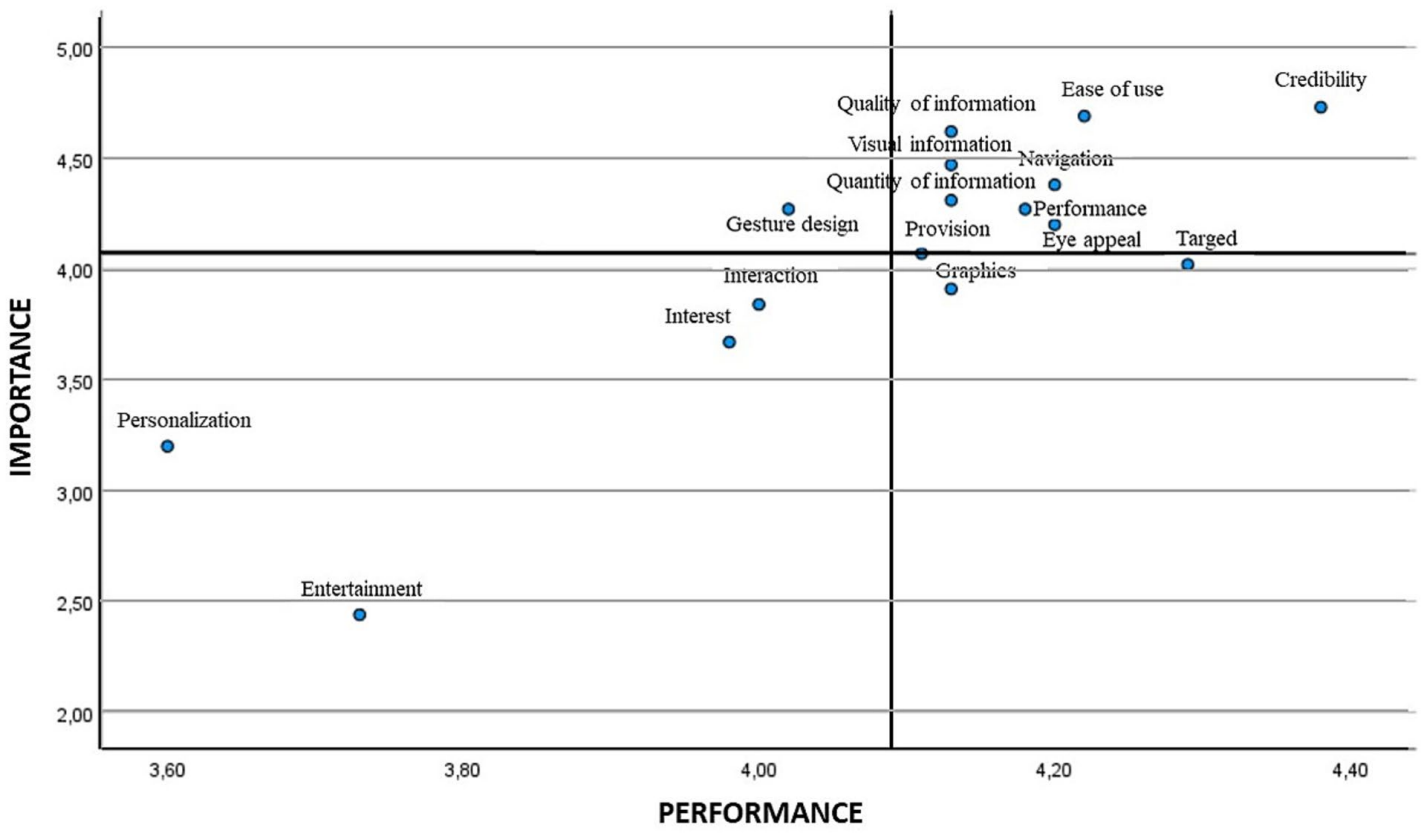
IPA analysis in the age range of 51–65.

When analysing the scatter plot, in the attributes that show low priority we find entertainment, interest, and personalisation/customisation in the four age ranges. The interaction attribute was also placed in this quadrant for the age range of 31–40 and over, and so was the graphics attribute in the age ranges of 17–30 and 41–50. The lowest age range 17–30 also placed eye appeal/visual appeal as a low priority. Finally, regarding attributes of high importance and low satisfaction, and therefore those on which attention must be focused, gesture design/gestural design appeared in all the age ranges, eye appeal/visual emerged in the age ranges of 31–40 and 41–50 years, and navigation in the youngest age range (17–30) (Figures 4–7).

### 3.4. Cluster analysis

The cluster analysis was determined according to the variable of recommending the fitness App to other people. Cluster 1, labelled “High Recommendation,” made up 71.3% of the sample, 77.9% females, 35.9% between 31–40 years, and represented the fitness App users who had a high intention of recommending the App to other people who could benefit from it (*M* = 4.40). Cluster 2 was designated “Low Recommendation” because the score of recommendation to other people shown by fitness App users was low (*M* = 2.61). This represented 28.7% of the sample. 62.3% were females, and 40.9% between 41–50 years old.

Table 6 shows the descriptions of the variables of perceived quality of the fitness App considering the IPA analysis according to the two cluster groups obtained. The results show two clear differences in accordance with the importance and the rating given by both groups. While the “High recommendation” group generally scores higher on all the factors and items in importance than the “Low recommendation” group, this is not the case for the items entertainment and interest. For both groups the most important item was credibility, followed by quality of information. Statistically significant differences were observed for the items performance, graphics and credibility (*p* < 0.05).

**Table 6:** Cluster analysis descriptive of IPA analysis.

Items	High recommendation		Low recommendation		U-Mann–Whitney	
	Imp.	Perf.	Imp.	Perf.	Imp.	Perf
*Commitment*	3.34	3.88	3.32	3.00	10574.0	4003.5***
Entertainment	**2.17**	**3.42**	**2.24**	**2.41**	10416.0	5938.5***
Interest	3.40	3.89	3.46	2.84	10421.5	5314.5***
Personalization/Customization	**3.28**	**3.69**	**3.28**	**2.97**	10652.5	7014.0***
Interaction	3.76	4.04	3.68	3.32	10386.5	6998.0***
Targed audience	4.07	4.34	3.96	3.48	10014.5	5390.0***
*Functionality*	4.40	4.24	4.23	3.40	9659.0	4687.5***
Performance	4.35	4.31	4.04	3.59	9212.0*	6675.0***
Ease of use	4.63	4.37	4.46	3.43	9894.5	5482.5***
Navigation	4.47	4.23	4.37	3.33	10193.5	5693.0***
Gesture design/Gestural design	4.17	4.05	4.06	3.23	10338.5	5872.0***
*Aesthetic*	4.05	4.19	3.94	3.15	9963.0	3519.5***
Provision/Disposal	4.03	4.24	4.23	3.37	9440.5	5796.5***
Graphics	3.97	4.14	3.62	3.23	8862.0**	5278.5***
Eye appeal/Visual appeal	4.15	4.17	3.97	2.87	9981.5	3560.5***
*Information*	4.52	4.34	4.37	3.60	9854.5	4157.5***
Quality of information	**4.65**	**4.38**	**4.51**	**3.65**	9983.0	5667.5***
Quantity of information/Amount of information	4.30	4.25	4.20	3.48	10595.5	5967.5***
Visual information	4.38	4.21	4.19	3.24	9754.5	4559.0***
Credibility	**4.76**	**4.52**	**4.58**	**4.03**	9551.5*	7295.5***

Highlights in bold; Imp, importance; Perf, performance. *p < 0.05, **p < 0.01, and ***p < 0.001.

On the other hand, the results of the perceived quality rating of the fitness App performance showed clearly lower scores than the importance, especially in the “low recommendation” group. In the “High recommendation” group the scores did not vary much from the importance rating. There were statistically significant differences in all the items according to recommendation in performance, while in importance these were only so in the items related to performance, graphics and credibility (*p* < 0.05).

## 4. Discussion

The aim of this study was to find out how sports users behave in terms of the importance and value they attach to the perceived quality of fitness Apps according to the consumer’s gender and age. To date, no studies have been found in the scientific literature that have analysed fitness Apps from the perspective of this method of analysis are in line with these two decision-making variables (65).

Aicher et al. (69) state that the IPA method has been used sparingly in sports research, where most studies that have evaluated perceived quality in the sports context have only focused on the evaluation of performance. Therefore, this work becomes one of the first studies to offer valuable information for fitness App developers and sport managers as they can learn what aspects consumers value best in a fitness App in order to increase its use, and possibly the users’ frequency of physical activity (11). A recent study found that according to importance, the most relevant factors for predicting fitness app intention to use were perceived enjoyment, followed by perceived system quality and perceived usefulness (58). For performance, the most relevant factors were also perceived enjoyment and perceived quality of system, while the third factor in this case was perceived ease of use.

Following the guidelines of Ferreira-Barbosa et al. (10) and Gómez-Ruiz et al. (70), this study has identified the aspects that fitness App users consider most important, as well as those that are most highly rated. In this way, developers could adapt the features and content of the fitness App according to the needs of the target user. Wang et al. (8) found that when comparing the current fitness app market offerings and the preferences of fitness app users, there is a mismatch in user perceptions. This implies that there is currently much room for improvement within the app market when it comes to developing specific apps that match user preferences and needs.

The results obtained in the total sample highlighted that users consider the credibility of the information they receive from the fitness App to be more important. In this line, Valcarce and Díez (20) obtained, to a certain extent, similar results in their study on adherence to sports services through fitness Apps, finding that the aspects most valued by users when using a fitness App occur when it collects accurate, meaningful and useful data. Fitness app developers should focus on the usability and quality of apps by creating online training courses on the use of technologies and apps (41).

Therefore, considering existing behavioural theories, the different studies determine that the usefulness of the App is the main factor when considering its use (10, 71–75). In other words, sports users seek continuous improvement of their fitness through challenges or goals that are set by the data they receive through the fitness App. For example, Oyibo and Vassileva (76) found that perceived usefulness and perceived aesthetics were highly correlated with users’ receptiveness to the persuasive features of a fitness App, which can be reaffirmed by considering that the aspect that scored lowest in the results of this study in both importance and rating was training. In other words, users are not looking for a fitness App to be fun and entertaining, but for it to be useful and to show accurate information about different parameters of their physical activity. In line with Zhang and Xu’s study (77), the entertainment of fitness Apps turned out not to be of great importance when using them, as users were not looking for fun, but rather to achieve health and fitness goals. In contrast, Zhu et al. (41) consider the promotion of games through gamification, as their results highlighted the role of fun when using fitness apps.

These data can provide usage behavioural clues as to what factors determine the perceived quality of fitness Apps to a greater or lesser degree by users. It is also important to keep in mind that both credibility and entertainment are subjective and difficult to measure (14). These factors can be key to consolidating the basis of what predisposes to the use of a fitness App and generates a higher perceived quality in its sports users (78). Considering gender, females attach greater relative importance to the quality of fitness app information than males (58).

Similar to the data obtained in the total sample, credibility scores the best and entertainment the worst when analysed by age group or, in the case of gender, in the female sample. However, although the male group rated credibility as the highest rated item, it was the quality of information that was the most important item for the male group. Females scored significantly higher on the factors of functionality, aesthetics and information than males. These results may be due to the fact that the females had been enrolled in the fitness centre for less time than the males, who had more experience but no significant differences in length of stay. These results are consistent with Lee and Cho’s (79) study in which users placed high importance on the credibility and quality of information in fitness Apps. Yet, male users tend to have a higher perceived usefulness of fitness apps than females and are less concerned about the use of fitness apps (80).

Furthermore, these researchers identified that credibility positively predicted the intention to continue using these Apps. This was supported by Zhang and Xu (77), who identified that users who employed fitness Apps rated credibility as one of the most important factors for them, although no gender distinctions were made. Another study showed that the perceived credibility of fitness Apps depended, to some extent, on the aesthetics they display to their users, even irrespective of their gender and age (81).

In this study, the variable most closely related to aesthetics was the visual appeal item. This aspect is of great importance for the users considering the total sample, although it did not receive a good rating, placing visual appeal in quadrant I of the IPA, where the focus of the work to be done to improve the App should be placed. These results are also reflected in the analysis according to gender, as for the female group visual appeal was placed in quadrant III of low priority.

With regard to age, it was observed that the most valued aspect in all the age groups was credibility. In turn, the quality of information was the second most highly rated aspect in all the groups except the oldest age group, 51–65 years, where ease of use was the second most highly rated aspect. On the other hand, among the aspects with the lowest ratings and importance was entertainment, which became less important as the user’s age increased. However, performance increased as the age range increased. Another aspect worth highlighting is the item of personalisation, which had the lowest score. Its behaviour is observed to decrease in importance and value from the youngest age group to the oldest age range. Personalisation is the aspect with the lowest performance for the 51–65 age group, behind entertainment.

As we have seen, age is a relevant factor when developing fitness Apps (30, 52). Older users attach greater importance to the accuracy of the information and the ease of use of the App than to the design and personalisation of the fitness App or entertainment. Therefore, the usability of the fitness App is more influenced by the perceived usefulness of the user than by the look and feel of the App. These results may also be justified to the extent that the time spent in the sports centre by the 51–65 years old group was significantly longer than the younger age group. In terms of age, older people are less interested in using fitness apps, perceive them to be less useful and have greater privacy concerns than younger people (30).

The cluster analysis showed the existence of two well-differentiated groups with respect to the dependent variable of recommending the fitness App to third parties. On the one hand, there is a high proportion of users with high intentions of recommending the fitness App as they consider that it can benefit these people. On the other hand, approximately three out of ten users consider that recommending the fitness App to others is not favoured. Within the literature, no studies have been identified that have conducted a cluster analysis considering the intention to recommend a fitness App. These results may corroborate to some extent the results of the IPA analysis on the satisfaction of the fitness App attributes placed mostly within quadrant II (65). Ultimately, a high perceived quality may be indicative of a high intention to recommend the fitness App.

In summary, agreeing with Aicher et al. (69), the results of the IPA analysis are useful and allow us to measure both the importance and the performance benefits to understand fitness app users’ attitudes towards fitness app attributes. Furthermore, the results show that with regard to the direction fitness apps are currently taking, they need to take into account the target audience they are aimed at. This would allow them to focus more on one aspect or another depending on the characteristics of the user, including the ability of the app itself to adapt to the behaviour and singularities of the person who is going to use it (70).

### 4.1. Limitations and future research

The main limitations encountered when carrying out this study were, firstly, the small sample size given that the fitness centres analysed are boutique fitness centres. On the other hand, we analysed the data extracted through a specific fitness App, without considering those that may be the most used by users and downloaded from Android or IOS shops. Another limitation is the lack of scientific literature on the subject. There are no similar previous studies that set out specific guidelines to follow and that provide data on the aspects studied to compare the results obtained with other similar studies. Finally, convenience sampling prevents the results of the study from being generalised. Furthermore, the sample obtained is distributed in an unbalanced way with respect to the higher proportion of females and a lower proportion of older adult participants. As for future lines of research, it is proposed that a study be conducted with a larger sample that allows for a better understanding of the fitness app user population. In future research, it is encouraged to continue applying this type of analysis to different apps in order to establish evidence. Stratified probability sampling should also be conducted to obtain similar proportions of the sample by gender or age. Finally, further study of fitness Apps usage behaviour in older adults is advocated. As indicated, most fitness Apps are targeted at younger adults, and few are personalised for older adults. In relation to the fitness App used, future studies need to be conducted with it to compare the data with those of the present study and corroborate the results and conclusions. On the other hand, it is necessary to carry out new studies with other apps to collect new data and establish the fundamentals of the usage behaviour of current and future older adults, as they belong to different technological generations.

## 5. Conclusion

In relation to the objective of this study, it is concluded that, regarding the usage behaviour of a fitness App, credibility is the most important and valued factor for users, regardless of their gender or age. The second factor was quality of information and the third ease of use. This is important as credibility was the highest rated and most important aspect in terms of gender and age. The cluster analysis corroborates the degree of satisfaction of the attributes, showing the existence of two groups according to the intention to recommend the fitness App, highlighting that around 70% have a high intention to recommend the use of the fitness App because of the benefits it can bring to other people. The findings show two groups in accordance with the perception of the fitness App. The high recommendation group scored higher on all factors and items in importance than the low recommendation group, except for the items entertainment and interest. For both groups the most important item was credibility, followed by quality of information. Also, the results showed significant differences for the items performance, graphics and credibility.

On the other hand, those responsible for the Fitbe fitness App should take into account the data obtained in this study to adapt to the needs demanded by their audience, in particular, to improve the gestural design and the visual appeal of the app. In conclusion, a fitness App that offers the user what they are looking for will allow them to have a high positive perceived quality and, therefore, a better experience during its use, which will result in greater satisfaction in this use. To this end, knowledge of the variables that influence fitness App usage behaviour will be key. Finally, the findings have shown that age is a factor to be borne in mind, as older users may feel less identified when using a fitness App.

### 5.1. Practical implications

Based on the results obtained in this study, the target audience of a fitness App must be taken into account, from the development to the content of the app. On the other hand, consumers are primarily looking for a credible, easy-to-use fitness App with a good quality of information and navigation. In contrast, sports users do not mind if it is not entertaining or if the presentation of the information helps them to meet their fitness and health goals. In the case of the Fitbe fitness App, the IPA analysis shows that they should focus efforts on improving the gestural design and visual appeal of the App.

There is a niche fitness market in older adults, so it is essential to take into account the needs and characteristics of this population in order to offer a fitness App adapted to them. According to the data obtained in this study, older adults gave the worst score to the personalisation of the Fitbe fitness App. In short, developers and managers of fitness Apps should consider carrying out an IPA analysis that shows the strengths and weaknesses of their Apps. In addition, an IPA analysis would allow them to better understand their users and their needs, being able to select the content shown in the App according to the registered profile, which is essential for fitness app managers in the quest to consolidate a greater number of customers in their services through technology.

## Data availability statement

The raw data supporting the conclusions of this article will be made available by the authors, without undue reservation.

## Author contributions

FM and JG-F: conception of the study and collected data. PG-R and SA-S: statistical analysis. MV-T and AB-G: literature review and writing the first draft of the manuscript. FM, JG-F, and PG-R: review and edition of the draft of the manuscript. All authors contributed to the article and approved the submitted version.

## Funding

This research was funded by the Junta de Andalucía, Regional Ministry of Economic Transformation, Industry, Knowledge and Universities (grant number AT 21_00031).

## Conflict of interest

The authors declare that the research was conducted in the absence of any commercial or financial relationships that could be construed as a potential conflict of interest.

## Publisher’s note

All claims expressed in this article are solely those of the authors and do not necessarily represent those of their affiliated organizations, or those of the publisher, the editors and the reviewers. Any product that may be evaluated in this article, or claim that may be made by its manufacturer, is not guaranteed or endorsed by the publisher.
